# Ethical and Psychosocial Issues Associated with Genetic Testing for Hereditary Tumor Predisposition Syndromes

**DOI:** 10.3390/healthcare13080880

**Published:** 2025-04-11

**Authors:** Mari Hachmeriyan, Mariya Levkova, Dinnar Yahya, Milena Stoyanova, Eleonora Dimitrova

**Affiliations:** 1 Department of Medical Genetics, Medical University Varna, 9000 Varna, Bulgaria; mariya.levkova@mu-varna.bg (M.L.); dinnar.yahya@mu-varna.bg (D.Y.); milena.stoyanova@mu-varna.bg (M.S.); 2 Laboratory of Medical Genetics, University Hospital “Sveta Marina”, 1 Hristo Smirnensky Str., 9000 Varna, Bulgaria; 3 Department of Oncology, Medical University Varna, 9000 Varna, Bulgaria; eleonora.dimitrova@mu-varna.bg

**Keywords:** ethics, hereditary cancer, tumor predisposition syndromes

## Abstract

Hereditary tumor predisposition syndromes (HTPSs) significantly increase the risk of developing various cancers, often at earlier ages than seen in the general population. The development and application of next-generation sequencing (NGS) has revolutionized the identification of individuals with HTPS, facilitating early diagnosis, personalized risk assessment, and tailored preventive strategies. However, the widespread implementation of genetic testing for HTPS presents complex ethical and psychosocial issues. This paper examines key ethical considerations surrounding genetic testing for HTPS, including the following: the distinct nature of genetic information and its implications for families; the challenges of informed consent amidst evolving genetic knowledge and direct-to-consumer testing; the complexities of predictive and presymptomatic testing, particularly in minors; and the implications of incidental findings. It further explores the critical issue of genetic discrimination, particularly concerning insurance, employment, and social stigmatization. This paper highlights the importance of balancing individual rights, such as autonomy and privacy, with familial responsibilities and the potential benefits of early detection and intervention. It also underscores the need for robust legal frameworks, comprehensive genetic counseling, and ongoing public education to address the ethical and psychosocial challenges associated with genetic testing for HTPS, with the ultimate goal of maximizing the benefits of genomic medicine while minimizing potential harms.

## 1. Introduction

Hereditary tumor predisposition syndromes (HTPSs), also widely known as hereditary cancer syndromes, are characterized by an increased risk of developing specific cancers, often at a younger age than typically seen in the general population. Routinely applied genomic diagnostic methods, such as next-generation sequencing (NGS), have created opportunities for early diagnosis, risk assessment, and crucially, personalized medicine approaches. Identifying the specific genetic basis of cancer risk allows for tailored treatment selection (e.g., targeted therapies like PARP inhibitors for individuals with *BRCA* mutations), personalized prevention strategies (such as decisions about prophylactic surgery), and individualized surveillance plans (determining the type and frequency of screening). The integration of these genetic tests into clinical practice raises complex ethical and psychosocial issues. As Van Rensselaer Potter [1], who introduced the term “bioethics” in 1970, noted, rapid scientific advances can potentially outpace due consideration of their moral and ethical implications. This observation remains profoundly relevant today. While ethical guidelines in research and medical practice have been implemented to provide adequate and clear ethical standards, care must be taken to ensure that these guidelines do not unduly hinder the development and responsible application of beneficial technologies [2]. Balancing innovation with ethical considerations is paramount in realizing the full potential of genomic medicine for individuals and families affected by HTPS.

## 2. Specificity of Genetic Information and Balancing Responsibility and Individual Rights

The unique nature of genetic information compared to other medical data raises ethical questions, particularly due to its “shared” nature among family members [3]. Although initially restrictive, normative documents, such as position statements from the American Society of Human Genetics (ASHG) [4] and the World Health Organization [5], have increasingly supported the disclosure of genetic information without patient consent in limited circumstances; however, the conditions for such disclosure often remain poorly defined [6]. Genetic risk information for medically actionable conditions offers potential benefits for early detection and prevention but can also complicate family relationships, for instance, by creating feelings of guilt in carriers or blame towards the side of the family the mutation originated from or through the unwanted disclosure of risk status to relatives who preferred not to know. Cascade genetic testing, which involves systematically offering genetic testing to biological relatives of an individual found to have a specific pathogenic variant to identify others who may also be at increased risk, is crucial for maximizing the preventative potential within families. Uptake in at-risk families is often below 50% [7], particularly in conditions like Hereditary Breast and Ovarian Cancer (HBOC) syndrome involving *BRCA1/2* mutations or Lynch syndrome, where structured cascade screening programs aim to identify affected relatives. Relatives may decline testing due to various reasons, including anxiety about the result, fear of genetic discrimination, potential impact on insurance, denial, or simply asserting their right not to know. Furthermore, the burden of contacting relatives, traditionally falling on the initially tested individual (the proband) although sometimes facilitated by healthcare professionals with patient consent, presents a significant barrier to the dissemination of risk information [8]. Still, the duty of care to inform relatives about potential genetic risks must be carefully balanced against an individual’s rights to privacy and autonomy in making decisions about their own health information.

## 3. Uncertainty in Genetic Testing

Genetic testing, particularly for HTPS, holds immense promise for proactive healthcare. However, a significant challenge lies in the inherent uncertainty associated with interpreting the results. For instance, discovering a pathogenic variant in a high-risk gene (e.g., BRCA1/2, PALB2) provides information about the likelihood of developing specific cancers or elevated risk compared to the general population [9]. However, for many moderate- or low-risk genes, testing often cannot definitively predict disease severity or the age of onset [10]. This limitation stems from complex factors such as incomplete penetrance, variable expressivity, modifier genes, and environmental and lifestyle factors influencing disease development and progression. While polygenic risk scores (PRSs), which aggregate the small effects of many common genetic variants (polymorphisms) across the genome to estimate an individual’s overall genetic susceptibility to a specific disease, are increasingly incorporated into risk prediction models alongside traditional risk factors, their clinical application is still evolving. Unlike tests for high-penetrance mutations in single genes (like *BRCA1*) that confer a large, relatively well-defined risk, PRSs assess a more generalized, probabilistic risk based on common variations. Consequently, their ability to accurately predict outcomes like breast cancer subtypes on a population-wide scale and their readiness for widespread clinical use still face challenges [11], although research is rapidly advancing.

This inherent lack of a precise genotype–phenotype correlation can significantly complicate the decision-making process for both patients and healthcare providers, particularly when considering preventive measures. For example, a woman with a pathogenic variant in BRCA1, BRCA2, or PALB2 faces a difficult choice regarding prophylactic mastectomy or oophorectomy. While these interventions can significantly reduce cancer risk, they can involve major surgery with potential physical and psychological consequences [12].

Moreover, our understanding of the human genome remains incomplete. Many genes and genetic variants currently lack established correlations with specific diseases. Most individuals receive results that are clinically uninformative, meaning either no pathogenic or likely pathogenic variants are identified in the genes tested, or only variants of uncertain significance (VUS) are found, for which the impact on disease risk is unknown. Notably, a study by Rao et al. [13] found no significant psychosocial harm among adults receiving negative genomic screening results for adult-onset hereditary conditions and only about 10% planned health-related behavior changes. In addition, ongoing research may reclassify variants of uncertain significance in the future, highlighting the dynamic and evolving nature of genetic knowledge and its impact on the interpretation of test results [14]. Professional societies like the American College of Medical Genetics and Genomics (ACMG) and the Clinical Genome Resource (ClinGen) provide guidelines on the interpretation and reporting of VUSs, acknowledging their limited clinical utility while emphasizing the need for periodic re-evaluation [15,16].

Therefore, pre-test and post-test genetic counseling plays a vital role. Counselors help individuals understand the limitations and uncertainties inherent in genetic testing, provide crucial support in navigating complex decisions, and assist individuals in weighing the potential benefits and risks considering their personal values and preferences [17,18].

## 4. Informed Consent in the Context of Oncogenetic Testing

Oncogenetic testing, with its profound implications for individuals and their families, necessitates a rigorous approach to informed consent. This process is further complicated by the sensitive nature of genetic information and its potential for broad use, including research. The General Data Protection Regulation (GDPR) (Regulation (EU) 2016/679), implemented by the European Union and effective since 2018 across the European Economic Area (EEA), provides a crucial framework for the protection of personal data, applying specifically to any information relating to an identified or identifiable natural person. It designates genetic data and health data as “special categories of personal data” under Article 9, affording them a higher level of protection.

In the context of oncogenetics, informed consent is not merely a formality but a dynamic process. The dialog should cover the purpose, procedures, potential benefits, and risks of the specific oncogenetic test, as well as the implications of the results for the individual and their family. It should also address issues of data storage, access, and potential future use. To facilitate participation, especially for geographically dispersed individuals, parts or all of the informed consent process can occur remotely (e.g., telephone, video conferencing, web-based methods). While web-based platforms offer feasibility for dynamic consent, their full utility requires further investigation [19].

Furthermore, to advance knowledge in oncogenetics and improve patient care, samples, genomic data, and health information collected during routine clinical care are often stored and used for research purposes. It is a common misunderstanding of current European data protection law that processing personal data is prohibited when consent does not have a lawful basis [20,21]. Article 9 (2) (j) of the GDPR allows exceptions to the prohibition of processing genetic data for such research, stating that processing is permissible when it is “necessary for archiving purposes in the public interest, scientific or historical research purposes, or statistical purposes”, subject to appropriate safeguards. In addition, Article 9 (4) allows Member States to introduce further conditions or limitations regarding the processing of genetic data. This has led to some variability in national regulations across the EU. For instance, some countries have explored using genomic data for research without explicit consent under certain conditions, potentially granting access to international bodies [22].

This debate connects directly to different models of consent for secondary data use. An opt-in system requires individuals to explicitly agree (give active consent) before their data can be used for a specific secondary purpose, such as research. Conversely, an opt-out system assumes consent for secondary use unless the individual actively refuses or objects (expresses dissent). The approval by European Parliamentarians of a proposal favoring an opt-in system for the secondary use of health data, including genetic data, in the proposed European Health Data Space (EHDS), provoked dissatisfaction from the European Society of Human Genetics (ESHG). This highlights the ongoing debate surrounding the balance between individual rights and control over data (emphasized by opt-in systems) and the potential benefits of facilitating broader data use in research (potentially enabled more easily by opt-out models allowed under specific GDPR provisions like Article 9 (2) (j), provided safeguards are met) [23].

## 5. Unsolicited/Secondary Findings

The application of next-generation sequencing (NGS) technologies, which encompass various high-throughput methods allowing for the simultaneous sequencing of millions or billions of DNA fragments, has profoundly impacted medical genetics, offering the potential to identify genetic information related to conditions other than the primary indication for testing, known as unsolicited or secondary findings (SFs). Although unsolicited findings (sometimes defined as findings in genes related to the tested indication but not the primary target) and secondary findings (in genes unrelated to the tested indication) can be distinguished, the broader term SFs is often used. Therefore, clinical genetics centers need to establish clear protocols, in accordance with ethical committee decisions and relevant guidelines, offering patients opt-in or opt-out choices regarding receiving SFs besides the initial diagnostic result [24]. The chance of identifying SFs in a gene panel depends on the scope of the panel and the specific genes included.

Van der Schoot et al. [25] found that SFs predisposing to medically actionable disorders affect a broader range of genes than those listed on the influential ACMG SF list, suggesting pre-defined lists may be too restrictive and that the evaluation of medical actionability may need to occur on a case-by-case basis. While the identification and disclosure of SFs depend on local policies, their study provides essential insights into the nature and odds of SFs in clinical exome sequencing (CES). CES specifically targets the sequencing of the exome—the protein-coding regions of the genome (approximately 1–2% of the total genome)—and is primarily used clinically for diagnosing individuals with suspected rare monogenic disorders or complex phenotypes unexplained by other testing or in cancer genomics to identify somatic and germline variants relevant to diagnosis, prognosis, or treatment. The American College of Medical Genetics and Genomics (ACMG) published 2013 recommendations for reporting SFs in clinical exome and genome sequencing, initially recommending the mandatory reporting of pathogenic variants in a predefined list of genes. The most recent recommendations (ACMG SF v3.2, 2023) expanded the list of genes associated with medically actionable conditions, including variants linked to numerous cancer predispositions [26].

Key points of contention surrounding SF reporting include potential infringement upon patient autonomy (if reporting is mandatory regardless of preference), the balance between potential benefits (early detection/prevention) and harms (anxiety, cost, and overtreatment) of disclosure, and the practical challenges of implementation in diverse clinical settings. Moreover, the legal framework governing the disclosure of genetic information to relatives varies considerably between jurisdictions, adding another layer of complexity.

## 6. Predictive and Presymptomatic Testing in Hereditary Cancer Syndromes

In the realm of hereditary cancer syndromes, genetic testing plays a crucial role in identifying at-risk individuals before the onset of symptoms. This testing generally falls into two categories: presymptomatic and predictive. While both aim to identify currently healthy individuals at risk, they differ significantly in their implications. Presymptomatic testing is used when a specific genetic mutation is known to inevitably cause a disease if the individual lives long enough (assuming complete penetrance); a positive result essentially confirms future disease development. Predictive testing, on the other hand, applies when a genetic variant is associated with an increased risk of developing a disease but does not guarantee its manifestation (due to factors like incomplete penetrance or variable expressivity). Predictive testing, therefore, deals with probabilities rather than certainties.

Regardless of whether testing is presymptomatic or predictive, the primary purpose is to maximize medical benefits. This includes enabling early detection through increased surveillance and screening, facilitating preventative measures such as prophylactic surgery or chemoprevention, and informing personalized treatment plans. When successful prevention or treatment is not possible, the information gained can still be valuable for life planning, making informed reproductive decisions, and allowing individuals to prepare for potential psychological and emotional challenges. However, it is crucial to acknowledge the potential downsides of such testing, even in healthy individuals. The psychological impact can be substantial, ranging from anxiety and depression to disruptions in family dynamics. Ironically, individuals who test negative for a known familial mutation might experience ‘survivor’s guilt’ [27], a complex emotional response.

Therefore, pre-test and post-test genetic counseling is paramount, providing comprehensive and accurate information about the specific syndrome, the genes involved, the limitations of testing (including the possibility of uninformative results or VUS), the potential psychosocial ramifications, and available support resources [28,29].

The landscape of genetic testing is becoming more widely available within clinical genetic and mainstream medical services. The potential consequences, which vary depending on the type of test (diagnostic, screening, presymptomatic, or predictive), must be considered prior to testing, recognizing that the field is constantly evolving. While considerable skepticism existed regarding the clinical utility of some direct-to-consumer (DTC) genetic tests by 2010, leading to critical policy recommendations from the ESHG [30], the field remains dynamic. Interest in DTC testing persists alongside evolving regulatory measures and professional guidelines aimed at ensuring responsible application [6]. Therefore, genetic susceptibility testing for individuals with relevant family histories can be beneficial when the information can effectively guide prevention or treatment, aligning with the principle of beneficence.

## 7. Genetic Testing in Asymptomatic Minors

Genetic testing in asymptomatic minors presents unique ethical and clinical challenges, particularly within the context of HTPS. For the purposes of this discussion, minors are defined as individuals who have not attained the legal age of majority to provide informed consent for healthcare decisions independently. In the specific context of oncogenetics, predictive genetic testing of asymptomatic minors should generally only be considered after the identification of a known familial pathogenic variant and primarily when preventive measures or surveillance begin in childhood, offering clear medical benefit during minority.

The European Society of Human Genetics (ESHG) published influential recommendations for genetic testing in asymptomatic minors in 2009 [31]. These guidelines emphasize respecting the minor’s future autonomy and the right to an open future, often advocating for deferring testing unless immediate medical benefits during childhood exist. The field of genetics has since undergone substantial advancements, warranting critical reappraisal and updates to existing recommendations. This is particularly relevant given discussions around potentially incorporating genomic sequencing into newborn screening programs [32] or even prenatal testing for adult-onset conditions [33].

In addition, parents may face challenges in disclosing positive test results to their children due to concerns such as the child’s age, developmental appropriateness, and the emotional burden involved. Further research is needed to understand these communication dynamics and how best to support parental decision-making and communication regarding childhood genetic testing for predisposition syndromes [34].

## 8. Genetic Discrimination in the Context of Hereditary Cancer Syndromes

Genetic discrimination poses a significant ethical challenge in medical genetics, particularly concerning hereditary cancer syndromes. It raises profound ethical concerns regarding equity, privacy, and the potential for stigmatization based on genetic information.

Internationally, there has been a growing recognition of the need to protect individuals from genetic discrimination. A landmark example is the US Genetic Information Nondiscrimination Act (GINA) of 2008, which prohibits discrimination in health insurance and employment based on genetic information. The American Society of Human Genetics (ASHG) has consistently advocated for the robust enforcement of the GINA and the enactment of similar legislation globally [35]. However, despite such legislation, recent studies confirm ongoing challenges. For example, individuals with Neurofibromatosis type 1 (NF1) experience higher rates of unemployment and sickness absence [36], often due to the condition’s variable manifestations which can include the growth of benign and malignant tumors, chronic pain, orthopedic issues, cognitive challenges, and the need for frequent medical appointments or surgeries. Furthermore, evidence suggests continued experiences of genetic discrimination faced by individuals with various hereditary conditions, alongside a limited awareness of legal protections [37].

Concerns about the adequacy of existing legal frameworks in protecting the privacy and security of genetic data may also lead patients to seek testing outside traditional healthcare systems (e.g., paying out of pocket or using direct-to-consumer services). This can result in sensitive genetic information being held by private companies, potentially outside the purview of healthcare regulations and electronic health record systems.

## 9. Conclusions

Genetic testing for hereditary tumor predisposition syndromes (HTPSs) offers significant potential for early cancer detection, risk management, and personalized medicine. However, this powerful technology presents complex ethical and psychosocial challenges. Balancing individual rights (such as autonomy and privacy) with familial responsibilities is crucial, especially given the shared nature of genetic information. The inherent uncertainties of genetic testing, including incomplete penetrance, variable expressivity, and variants of uncertain significance, necessitate comprehensive pre-test and post-test genetic counseling. Incidental or secondary findings further complicate clinical management and patient decision-making.

While legal frameworks like the GDPR in Europe and the GINA in the US exist to protect genetic information and prevent discrimination, gaps and enforcement challenges remain, particularly regarding life insurance, long-term care insurance, and disability insurance in some jurisdictions, as well as potential social stigmatization. The rise in direct-to-consumer genetic testing also requires ongoing regulatory attention and adaptation.

Moving forward, robust legal protections against genetic discrimination, comprehensive public education about genetics and its implications, and specialized training for healthcare professionals involved in ordering or interpreting these tests are essential. A continued focus on psychosocial research investigating the experiences of individuals and families undergoing testing is also critical.

Realizing the full benefits of HTPS testing demands a commitment to ethical principles, ongoing multidisciplinary dialog, and proactive approaches to address evolving challenges. This will help ensure that individuals and families can benefit from the advances in genomic medicine while safeguarding their rights and well-being. Addressing these interconnected ethical, psychosocial, and practical considerations is therefore fundamental for the responsible integration of genomic technologies into current and future healthcare, ultimately shaping how we deliver equitable and effective personalized care in an era of genomic medicine.

## Data Availability

No new data were created or analyzed in this study. Data sharing is not applicable to this article.
